# Dual non-contiguous peptide occupancy of HLA class I evoke antiviral human CD8 T cell response and form neo-epitopes with self-antigens

**DOI:** 10.1038/s41598-017-05171-w

**Published:** 2017-07-11

**Authors:** Ziwei Xiao, Zhiyong Ye, Vikeramjeet Singh Tadwal, Meixin Shen, Ee Chee Ren

**Affiliations:** 1 Singapore Immunology Network, Agency for Science, Technology and Research (A*STAR), 8A Biomedical Grove, #03-06 Immunos, Singapore, 138648 Singapore; 2 Department of Microbiology & Immunology, Yong Loo Lin School of Medicine, National University of Singapore, 5 Science Drive 2, Singapore, 119260 Singapore

## Abstract

Host CD8 T cell response to viral infections involves recognition of 8–10-mer peptides presented by MHC-I molecules. However, proteasomes generate predominantly 2–7-mer peptides, but the role of these peptides is largely unknown. Here, we show that single short peptides of <8-mer from Latent Membrane Protein 2 (LMP2) of Epstein Barr Virus (EBV) can bind HLA-A*11:01 and stimulate CD8^+^ cells. Surprisingly, two peptide fragments between 4–7-mer derived from LMP2_(340–349)_ were able to complement each other, forming combination epitopes that can stimulate specific CD8^+^ T cell responses. Moreover, peptides from self-antigens can complement non-self peptides within the HLA binding cleft, forming neoepitopes. Solved structures of a tetra-complex comprising two peptides, HLA and β2-microglobulin revealed the free terminals of the two peptides to adopt an upward conformation directed towards the T cell receptor. Our results demonstrate a previously unknown mix-and-match combination of dual peptide occupancy in HLA that can generate vast combinatorial complexity.

## Introduction

Recognition of viral antigens by CD8^+^ T cells requires successful folding of the major histocompatibility class I (MHC-I) molecule and β2-microglobulin (β2 m) with peptides typically 8- to 10-mer in length^1–3^. The peptide binding groove of MHC-I molecule contains six binding pockets, A-F, which span and accommodate the binding peptide from the N-terminal to C-terminal^4^. A minimum 8-mer peptide is required for structural stability of the peptide-human leukocyte antigen (pHLA) tri-complex that is contributed by two amino acid residues of the peptide that serve as “anchors”, one that fits in pockets A/B and the other in pocket F^4–6^.

In recent years, non-canonical lengths of peptides ranging from 11- to 16-mers in HLA are more frequently reported and increasingly recognized to contribute towards the MHC-I-restricted peptide repertoire^7^. On the other hand, it has been presumed that peptides of <8-mer do not play a significant role in the activation of CD8^+^ T cells, as they do not possess the two anchor residues required for stable peptide occupancy in HLA and are degraded in the cytosol^8^. Indeed, the two anchor residues requirement is noted even with a 5-mer MUC1 peptide binding to murine H-2K^b^ but not a 4-mer peptide with only one anchor residue^9^. However, as two-thirds of the breakdown products of standard proteasome are less than 8-mer in length and only less than 15% of the breakdown products fall within the 8- to 10-mer range^10, 11^, it remains to be examined if peptides <8-mer are immunogenic and able to stabilize the pHLA complexes.

Epstein-Barr virus (EBV) is a persistent virus carried by more than 90% of the world population^12^. Given its ability to persist as a latent infection, EBV serves as a good viral model for the examination of antigen-experienced CD8^+^ T cells specific for eliminated and/or latent viruses. Here, we utilized a well-characterized HLA-A*11-restricted EBV LMP2_(340–349)_ peptide sequence (SSCSSCPLSK) that elicits a strong cytotoxic T lymphocyte (CTL) response^13–15^ to assess the immunogenicity of peptides <8-mer. In addition, we also examined the ability of combinations of two non-canonical truncated peptides to stabilize the pHLA complex as well as to elicit a specific CD8^+^ T cell response. Our results demonstrate that peptides <8-mer harbouring a single anchor residue as well as combinations of two truncated peptides, are capable of stabilizing pHLA complexes and evoking an antigen-specific CD8^+^ T cell response. More importantly, the solved crystal structures of a tetra-complex form by two peptides, HLA heavy chain and β2 m light chain, as well as the detection of CD8^+^ T cells specific to neoepitopes in a healthy human individual provided evidences for patterns of non-canonical peptide occupancy in HLA that can activate T cells to confer protection or contribute to pathologies.

## Results

### Peptides with single anchor residue recall CD8^+^ T cell response

**Figure 1:**
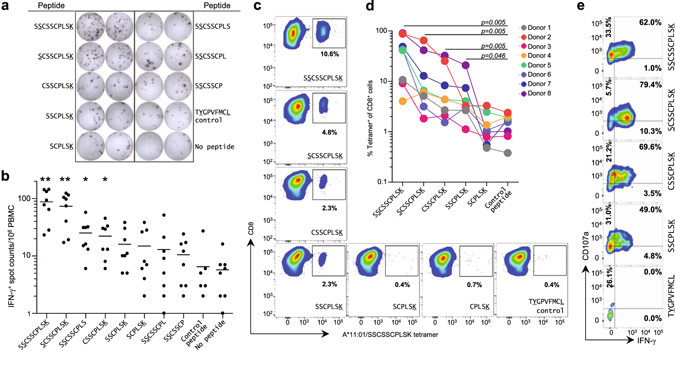
Antigen-specific CD8 T cell response to peptides <8mer. (**a**) PBMCs stimulated with SSCSSCPLSK as well as its C-terminal and N-terminal short peptides were assayed by ELISpot. Data from a representative HLA-A*11:01 homozygous individual is shown. TYGPVFMCL peptide (HLA-A*24 restricted) served as the control. (**b**) Counts of IFN-γ secreting spots from HLA-A*11:01 individuals (n = 8) represented as dot plot. Positive response defined as > 2 times negative control value and > 15 SFU/ 10^6^ PBMCs. Length of peptide affects T cell response (Cochran’s Q test, *p* = *0.00495*), statistically significant pairwise comparisons are indicated (McNemar post-test; **p < *0.01*; *p < *0.05*). (**c**) FACS plots from a representative HLA-A*11:01 homozygous donor’s 14-day PBMC culture show detection of T cells by A*11:01/SSCSSCPLSK tetramer following stimulation with SSCSSCPLSK, SCSSCPLSK, CSSCPLSK or SSCPLSK. PBMC cultures stimulated with irrelevant peptide control, HLA-A*24-restricted EBV peptide (TYGPVFMCL), were similarly stained with A*11:01/SSCSSCPLSK tetramer to serve as a negative control. (**d**) Line graph depicting A*11:01/SSCSSCPLSK tetramer-positive CD8^+^ cell percentages in eight A*11:01 positive individuals. Positive response defined as > 2 times control peptide value and more than 1% of the CD8^+^ cells. Length of peptide affects percentage of double-positive T cells (Cochran’s Q test, *p* < *0.001*), statistically significant pairwise comparison with negative control are indicated (McNemar post-test). (**e**) FACS plots from a representative HLA-A*11:01 homozygous donor’s 14-day PBMC culture show CD8 and A*11:01-SSCSSCPLSK tetramer double-positive cells expressed intracellular IFN-γ and surface CD107a after re-stimulation with SSCSSCPLSK, SCSSCPLSK, CSSCPLSK or SSCPLSK.

To determine whether non-canonical short peptides are immunogenic, full-length and truncated versions ranging from 6- to 10-mer starting from either N- or C-terminal of the HLA-A*11-restricted EBV LMP2_(340–349)_ peptide sequence (SSCSSCPLSK) were generated for use in an *in-vitro* peptide challenge assay. Peripheral blood mononuclear cells (PBMCs) from eight HLA-A*11:01 positive healthy human donors were incubated with these EBV LMP2 peptides and evaluated by IFN-γ ELISpot assay. Positive recall responses were observed not only with the native 10-mer full-length and 9-mer peptides but also with the 8-mer truncated sequence (CSSCPLSK) that possesses only one anchor residue (Fig. 1a,b).

In order to better assess the CD8^+^ T cell response quantitatively, HLA-A*11:01/SSCSSCPLSK tetramers were generated and used to stain 14-day peptide stimulation PBMC cultures from eight HLA-A*11:01 positive healthy human donors. Consistent with the ELISpot results, the 8-mer truncated sequence (CSSCPLSK) was able to elicit an antigen-specific CD8^+^ T cell response in the 14-day PBMC cultures that is significantly higher than that by the control peptide (*p* = *0.005*) (Fig. 1c,d). Interestingly, the results also revealed the ability of the 7-mer truncated sequence (SSCPLSK) to evoke a higher antigen-specific CD8^+^ T cell response as compared to the control peptide. This was overall significant for all donors (*p* = *0.046*), but due to inter-individual variability, the level of response was at the same level as compared to the control peptide for certain individuals such as Donor 2 and 3 (Fig. 1d). These demonstrated that the ability of non-canonical short peptides, such as CSSCPLSK and SSCPLSK peptides that contain only the PΩ anchor, to evoke recall CD8^+^ T cells is a common event in many HLA-A*11 individuals. Moreover, CD8^+^ T cells from these peptide challenge cultures were found to express the activation markers CD107a^16^ and IFN-γ^17^ (Fig. 1e); thus taken together, these data provide clear evidence that non-canonical short peptides are functional as immuno-stimulatory ligands for CD8^+^ T cells.

### Increased diversity in TCRVβ family usage elicited by shorter peptides

**Figure 2:**
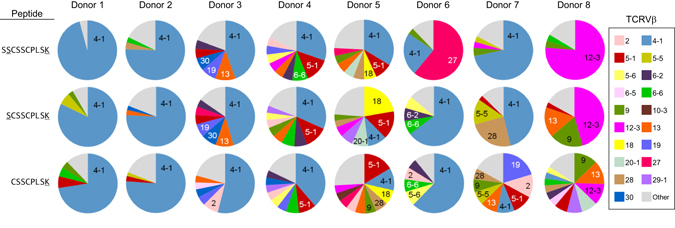
TCRVβ usage in CD8 and tetramer double-positive T cells from A*11:01 donors following different peptide stimulation. T-cell receptor Vβ (TCRVβ) usage in A*11:01/SSCSSCPLSK tetramer positive T cells stimulated with different lengths of SSCSSCPLSK peptide in 14-day culture of PBMCs from eight HLA-A*11:01 positive individuals.

To understand in greater detail the nature of CD8^+^ T cell responses to non-canonical short peptides, PBMCs post-peptide stimulation were stained with HLA-A*11:01/SSCSSCPLSK tetramer as well as TCRVβ (T-cell receptor variable beta) antibody panel. Thereafter, tetramer positive cells were analysed for their TCRVβ usage. Interestingly, different HLA-A*11:01 positive individuals utilized the same Vβ4–1 family as the main recognition specificity for the three peptides with decreasing lengths (SSCSSCPLSK, SCSSCPLSK and CSSCPLSK) (Fig. 2). This is consistent with a recent study that a CD8^+^ T cell clone utilizing TCRVβ4-1 has high avidity for the HLA-A*11-restricted SSCSSCPLSK^15^. As the original peptide gets shorter, there is a pattern of increased diversity in the usage of TCRVβ families (Fig. 2, Supplementary Table 1). With the observation that shorter C-terminal peptide (CSSCPLSK) with single anchor residue was able to elicit a pattern of TCRVβ usage different from those seen with the full length 10-mer peptide, this suggests that a more diverse pool of CTLs may be involved in the immune response to foreign antigens than what we would expect from stimulation by peptides defined by having two anchor residues.

### Single truncated peptides stabilize pHLA complexes of different alleles

**Figure 3:**
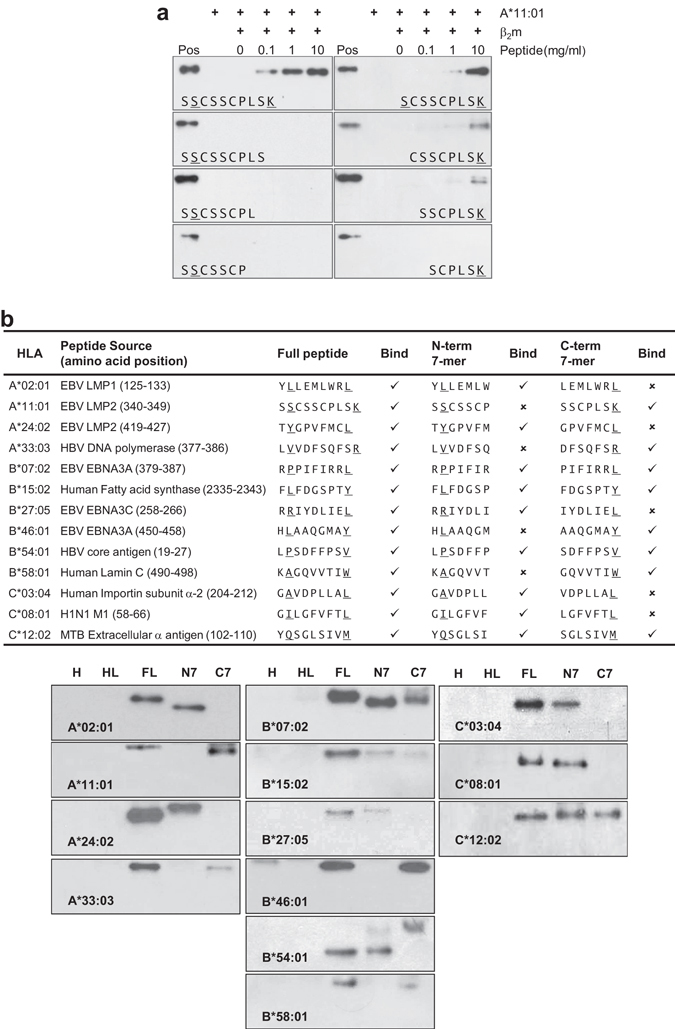
Stabilization of pHLA complexes by single non-canonical short peptides. (**a**) Stabilization of pHLA complexes formed by HLA-A*11:01 heavy chain and β2 m with HLA-A*11-restricted SSCSSCPLSK or its C-terminal truncated peptides (SCSSCPLSK, CSSCPLSK and SSCPLSK) in a dose-dependent manner (0, 0.1, 1 and 10 mg/ml) as determined by native western blot with W6/32 antibody. pHLA complex of HLA-A*11:01 heavy chain and β2 m refolded with 10 mg/ml of full-length SSCSSCPLSK peptide served as the positive control (Pos). HLA-A*11:01 heavy chain and HLA-A*11:01 heavy chain with β2 m served as the negative controls. (**b**) Tabulation of MHC class I molecules refolding *in-vitro* with N-terminal (N-term) 7mer peptide (P2 anchor underlined) and/or C-terminal (C-term) 7mer peptide (PΩ anchor underlined) as determined by native western blot with W6/32 antibody shown in the lower panels. Presence of bands detected by W6/32 antibody in the native western blot indicates the presence of stabilized pHLA complexes. Due to the difference in overall charge-to-mass ratio of the different pHLA complexes formed by different peptides and peptide lengths, a variation in the migration rates of the different pHLA complexes in the native PAGE gels can be observed. For each indicated HLA allele, heavy chain (H), heavy and β2 m light chain (HL) served as the negative controls while pHLA complex with full peptide (FL) served as the positive control for each western blot panel. EBV, Epstein-Barr Virus; HBV, Hepatitis B Virus; MTB, *Mycobacterium tuberculosis*.

To determine the binding ability of these short peptides to HLA, we performed *de novo* refolding of the HLA heavy chain, β2 m with individual peptides and examined the stability of the pHLA complexes with native western blot using W6/32 antibody. The refolding results showed that truncation of the PΩ (C-terminal) anchor (i.e. SSCSSCPLS, SSCSSCPL and SSCSSCP) abrogated binding, while progressive truncation of amino acids from the N-terminal was not deleterious to binding even when the P2 anchor was absent (i.e. CSSCPLSK and SSCPLSK) (Fig. 3a).

To further demonstrate that HLA alleles other than HLA-A*11:01 can bind non-canonical short peptides, we selected common HLA alleles including A*02:01, A*24:02, A*33:03, B*07:02, B*15:02, B*27:05, B*46:01, B*54:01, B*58:01, C*03:04, C*08:01 as well as C*12:02 and tested them using 7-mer truncated peptides that start from either the N-terminal or C-terminal thus ensuring that they bind with only one amino acid anchor residue. Intriguingly, we observed that these common HLA alleles were able to bind at least one, if not both of the N-terminal 7-mer containing the P2 anchor and the C-terminal 7-mer containing the PΩ anchor of the full-length peptide (Fig. 3b). As such, this capacity appears to be relatively widespread among many HLA alleles, and that with the right allele and its cognate peptide ligand, non-canonical binding of peptides <8-mer can occur.

### Stable pHLA complexes formed by two non-canonical peptides are immunogenic

**Figure 4:**
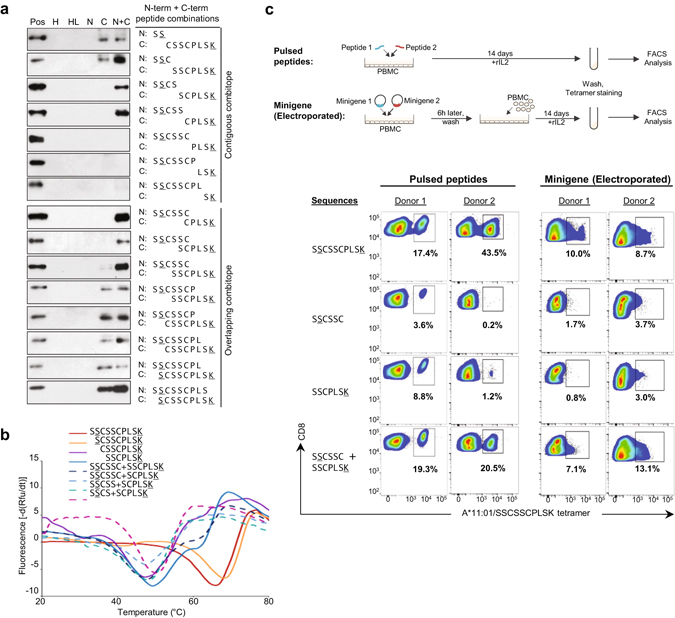
Stabilization of pHLA complexes by combinational non-canonical short peptides. (**a**) Stabilization of pHLA complexes formed by HLA-A*11:01 and β2 m with combination epitopes (combitopes) of short C-terminal (C) and N-terminal (N) SSCSSCPLSK peptides. Refolding with both C- and N-terminal SSCSSCPLSK peptides (N + C) generates combination of contiguous peptides (Contiguous combitope) as well as combination of peptides with increasing number of overlapping amino acids (Overlapping combitope). Heavy chain (H), heavy and β2 m light chain (HL) served as the negative controls while pHLA complex with SSCSSCPLSK (pos) served as the positive control for each western blot panel. (**b**) Real time PCR thermofluor measurement for pHLA complexes formed by HLA-A*11:01 heavy chain and β2 m with SSCSSCPLSK (Tm = 68.1 °C), SCSSCPLSK (Tm = 69.7 °C), CSSCPLSK (Tm = 49.4 °C), SSCPLSK (Tm = 50.0 °C), SSCSSC + SSCPLSK (Tm = 49.4 °C), SSCSSC + SCPLSK (Tm = 48.7 °C), SSCSS + SCPLSK (Tm = 49.7 °C) and SSCS + SCPLSK (Tm = 50.3 °C). (**c**) Workflow for 14-day PBMC cultures following peptide pulsing and minigene electroporation is shown. Truncated peptides SSCSSC and SSCPLSK were introduced in combination or singly in the form of pulsed peptides or electroporated minigenes constructs. Recall CD8 T cell responses were detected by A*11:01/SSCSSCPLSK tetramer staining of 14-day PBMC cultures in two different HLA-A*11:01 donors.

With peptides <8-mer forming stable pHLA complexes, the question arises as to whether another peptide can occupy the available binding cavity space. This is tested by using two separate peptides that complement each other to form the original full-length peptide (SS + CSSCPLSK; SSC + SSCPLSK; SSCS + SCPLSK etc.) to refold with the HLA heavy chain and β2 m. The results showed that a combination epitope (“combitope”) generated from 4-mer with 6-mer (SSCS + SCPLSK) and 5-mer with 5-mer (SSCSS + CPLSK) peptides could form stable pHLA complexes in contrast to the individual 4-mer, 5-mer or 6-mer which were unable to do so on their own (Fig. 4a). Thus the provision of a peptide anchor residue at the N-terminal (docking with B-pocket of HLA) and C-terminal (docking with F-pocket of HLA) by two separate peptides can successfully complement and confer structural stability to the pHLA complex.

As proteasomes generate overlapping sets of peptide fragments from the same protein^18^, we explored whether peptides with overlapping sequences are able to form stable pHLA complexes. Indeed, peptide occupancy was not restricted to contiguous peptide sequences as two peptides that overlap at one residue (SSCSSC + CPLSK), two residues (SSCSSC + SCPLSK) or three residues (SSCSSC + SSCPLSK) were also permissible (Fig. 4a, Supplementary Fig. 1a). The generation of stable pHLA complexes is not limited to combitopes from the SSCSSCPLSK peptide, as contiguous and overlapping combitopes from HLA-A*11-restricted ATIGTAMYK peptide (EBV BRLF1_134–142_)^19^ could similarly stabilize pHLA (Supplementary Fig. 1b).

To further evaluate the stability of the pHLA complexes formed by different peptide length and peptide combinations, we performed thermal stability assay on selected pHLA complexes. The results for the pHLA complexes demonstrated that the longer peptides with two functional peptide anchor residues had higher melting temperatures (Tm) (Tm = 68.1 °C for SSCSSCPLSK and Tm = 69.7 °C for SCSSCPLSK) and are thus more stable than those formed with only one anchor residue (Fig. 4b). pHLA complexes of the various combitopes generally had melting temperatures similar to that of the complexes formed by shorter peptides with one anchor residue (Tm ranging between 48.7–50.3 °C) (Fig. 4b). This observation is consistent with the preference of two anchor residues for binding to HLA molecules^20, 21^, but at the same time demonstrates that single anchor residue as well as a combination of two shorter peptides with an anchor each, would suffice for binding, albeit with lower stability.

We next assessed the functional capabilities of such dual peptides in combination. Remarkably, when tested in pulsed peptide challenge assays, these truncated peptides were able to stimulate higher antigen-specific CD8^+^ T cell responses in PBMCs incubated with SSCSSC + SSCPLSK in combination (19.3% in Donor 1 and 20.5% in Donor 2) compared to stimulation by SSCSSC or SSCPLSK alone (3.6% and 8.8% respectively in Donor 1; 0.2% and 1.2% respectively in Donor 2) (Fig. 4c). Other peptide challenge combinations (SSCS + SSCPLSK; SSCSS + SCPLSK; SSCSS + SSCPLSK; SSCSSC + CPLSK) were also able to elicit such synergistic antigen-specific CD8^+^ T cell responses (Supplementary Fig. 2a,b). Furthermore, our results have also demonstrated that such synergistic antigen-specific CD8^+^ T cell responses were not limited to combinations of peptides arising from the SSCSSCPLSK peptide. Dual peptide combination (ATIGTA + GTAMYK) from A*11-restricted ATIGTAMYK peptide was also able to stimulate a higher percentage of antigen-specific CD8^+^ T cell response in PBMCs incubated with the combination of peptides as compared to single peptides alone (Supplementary Fig. 3).

To demonstrate that truncated peptides originating endogenously are also able to form combitope capable of stimulating specific CD8^+^ T cells, SSCSSC-minigene and SSCPLSK-minigene expression vectors were constructed and electroporated into donor PBMCs (outlined in Fig. 4c). Synergistic specific CD8^+^ T cell responses were detected in the cells receiving both SSCSSC-minigene and SSCPLSK-minigene (7.1% in Donor 1 and 13.1% in Donor 2) compared to cells receiving SSCSSC-minigene or SSCPLSK-minigene alone (1.7% and 0.8% respectively in Donor 1; 3.7% and 3.0% respectively in Donor 2) (Fig. 4c). This provides strong evidence that complementation of truncated peptides is a physiologically relevant event and generates a pHLA tetra-complex capable of activating T cells. Notably, while the combination epitopes formed by two separate peptides may not be identical to that of the original 10-mer, TCRVβ usage analysis showed that Vβ4–1 was still dominant but greater diversification of TCRVβ usage was observed (Supplementary Fig. 4).

To further examine whether HLA alleles other than A*11:01 are also able to accommodate combitopes generated by two separate truncated peptides, refolding assays were performed with A*02:01; A*24:02; A*33:03 and B*46:01. Indeed, stable pHLA complexes were easily formed by these HLA alleles with their respective restricted peptides^22–25^: A*02:01 (YLLEML + MLWRL), A*24:02 (RYSIF + IFFDYM; RYSIFF + FDYM), A*33:03 (ASGK + QMWQAR) and B*46:01 (HLAAQ + QGMAY) (Supplementary Fig. 5a). In addition, when tested for their ability to stimulate PBMCs from donors with the specific HLA alleles, these combinations of dual truncated peptides elicited stronger IFN-γ ELISpot responses compared to that by individual truncated peptides alone (Supplementary Fig. 5b). Taken together, these data reveal an entirely new layer of immune response mechanism that is made possible by peptide occupancy consisting of two non-canonical short fragments.

### Combination of self and non-self peptides evoked CD8^+^ response

**Figure 5:**
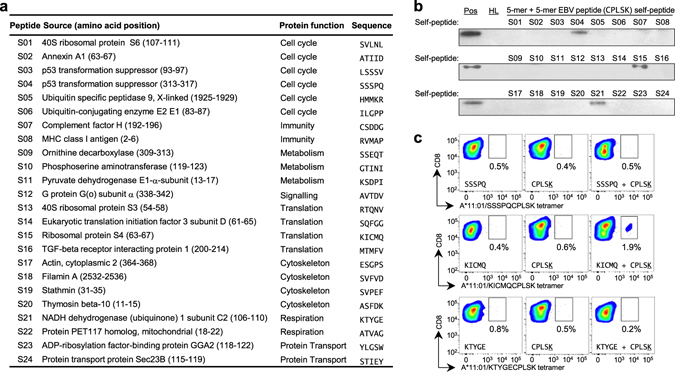
Presence of T cells activated by combination of short peptides from self and non-self antigens. (**a**) List of N-terminal 5mer peptides from endogenous self-protein known to bind HLA-A*11:01 that were used for refolding screening. (**b**) Native western blot screening results indicate formation of stable pHLA complexes from refolded HLA-A*11:01 heavy chain, β2 m and EBV LMP2 C-terminal 5mer peptide (CPLSK) together with three self-peptides (S4:SSSPQ, S15:KICMQ and S21:KTYGE). (**c**) Detection of CD8^+^ T cells that recognize self and non-self combinatorial epitope in 14-day PBMC culture of a representative HLA-A*11:01 homozygous individual after stimulation with endogenous self 5mer N-terminal peptides (S4, S15 and S21), CPLSK peptide or the combinatorial self and non-self peptides.

As two separate peptides can simultaneously occupy the HLA binding groove, the sources of these peptides can be potentially highly diverse. One important source of utmost interest to explore is that of self-antigens. To put this to the test, a panel of 24 self-antigens that can bind to HLA-A*11:01 was collated^26^ and the N-terminal 5-mer fragments were synthesized (Fig. 5a). These peptides were individually folded in combination with the EBV LMP2 C-terminal 5-mer (CPLSK). The results revealed that 5-mer peptides from p53 (SSSPQ), ribosomal protein (KICMQ) and subunit C2 of NADH dehydrogenase (KTYGE) were able to complement CPLSK to form stable pHLA complexes (Fig. 5b, Supplementary Fig. 6). To investigate whether these neo-epitopes are present, SSSPQ + CPLSK, KICMQ + CPLSK and KTYGE + CPLSK peptide combinations were used to stimulate PBMCs from normal healthy donors. Interestingly, of the three self-nonself peptide combinations, KICMQ + CPLSK was able to stimulate a specific CD8^+^ T cell response (Fig. 5c). The response of 1.9% of CD8^+^Tetramer^+^ cells, compared to 0.4% from KICMQ peptide stimulation alone and 0.6% from CPLSK peptide stimulation alone, is from a healthy subject. The results suggest that it is possible that such T cells arose by accident after a virus infection and may not cause any immediate pathology, but over time, they may become the precursors to an expanded population of cells that have auto-reactive potential.

### Crystal structures of pHLA tri- and tetra-complexes

**Figure 6:**
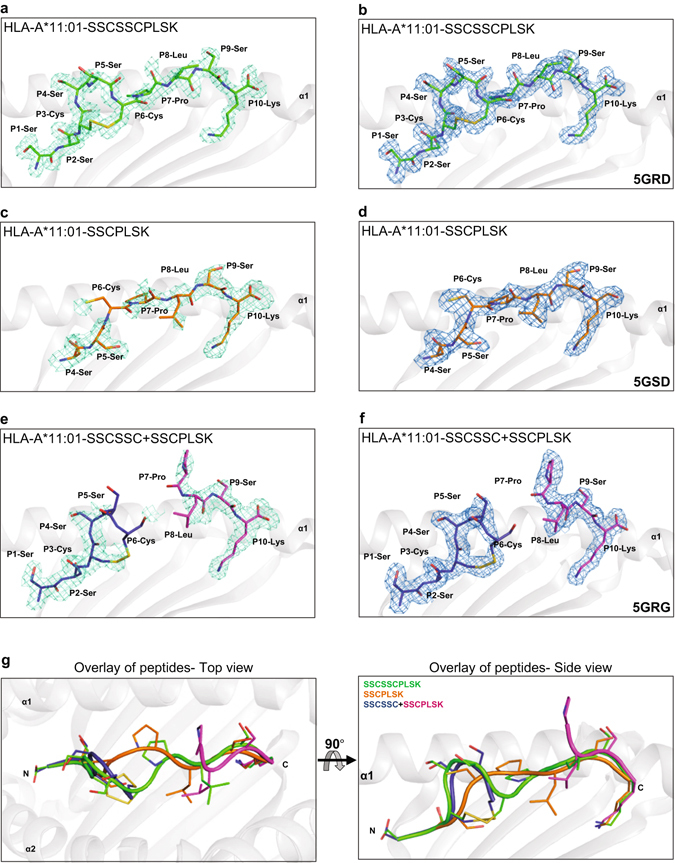
Electron density maps and comparison of SSCSSCPLSK, SSCPLSK and SSCSSC + SSCPLSK peptides bound to HLA-A*11:01. Omit maps *(F*_*o*_ *-F*_*c*_) at 2.5 σ in green mesh and *2F*_*o*_ *-F*_*c*_ maps at 1 σ in blue mesh after final refinement for each epitope in complex with HLA-A*11:01 (grey) are shown respectively in (**a**) and (**b**) for SSCSSCPLSK (green; PDB code: 5GRD); (**c**) and (**d**) for SSCPLSK (orange; PDB code: 5GSD); as well as (**e**) and (**f**) for SSCSSC + SSCPLSK (dark blue and magenta; PDB code: 5GRG). The peptide residues are represented in sticks. (**g**) The three peptides in the cleft of HLA*11:01 molecules were overlaid and compared. The left panel shows the top view of three peptides, SSCSSCPLSK (green), SSCPLSK (orange) and SSCSSC + SSCPLSK (dark blue and magenta) bound to HLA-A*11:01 molecule obtained by aligning only the Cα atoms. The right panel shows the side view of the overlap of the three peptides and the α2 domain of HLA-A*11:01 molecule is removed for clarity purpose. N- and C-termini of the peptides are labelled.

To further verify that two separate peptides can simultaneously occupy the single antigen binding cavity of MHC-I, we generated protein crystals comprising HLA-A*11:01 folded with SSCSSCPLSK with contiguous P2 and PΩ anchors (PDB code 5GRD, 1.8 Å), SSCPLSK with PΩ anchor only (PDB code 5GSD, 2.3 Å) and SSCSSC + SSCPLSK with non-contiguous P2 and PΩ anchors respectively (PDB code 5GRG, 1.9 Å) (Table 1, Fig. 6).

**Table 1:** Data collection and refinement statistics.

	HLA-A*11:01-SSCSSCPLSK^a^ (PDB Code: 5GRD)	HLA-A*11:01-SSCPLSK^a^ (PDB Code: 5GSD)	HLA-A*11:01-SSCSSC + SSCPLSK^a^ (PDB Code: 5GRG)
Data collection			
Space group	P2_1_2_1_2_1_	P2_1_2_1_2_1_	P2_1_2_1_2_1_
Cell dimensions			
*a*, *b*, *c* (Å)	54.42, 67.80, 115.30	55.35, 67.65, 115.60	55.25, 67.43, 115.02
a, b, γ (°)	90, 90, 90	90, 90, 90	90, 90, 90
Resolution (Å)	23.84–1.80 (1.86–1.80)^b^	58.38–2.30 (2.40–2.30)	58.17–1.94 (2.01–1.94)
R_merge_ (%)	3.8 (14.9)	10.9 (72.2)	5.7 (37.4)
*I*/*sI*	24.50 (6.85)	12.68 (2.07)	14.20 (2.50)
Completeness (%)	96.7 (96.6)	99.7 (100.0)	99.7 (99.9)
Redundancy	3.0 (3.0)	6.9 (4.7)	4.5 (4.6)
Refinement			
Resolution (Å)	58.44–1.80 (1.86–1.80)	39.98–2.30 (2.40–2.30)	30.00–1.94 (2.01–1.94)
No. reflections	38, 953	19, 869	30, 594
R_work/_ R_free_	17.6/22.6	19.5/27.3	18.9/24.3
No. atoms			
Protein	3112	3081	3073
Peptide	67	48	67
Glycerol	12	30	24
Water	413	258	256
B-factors (Å^2^)			
Protein	23.1	31.0	37.2
Peptide	18.8	49.8	58.5
Glycerol	38.4	46.8	49.5
Water	31.8	43.8	43.9
r.m.s.d.			
Bond lengths (Å)	0.019	0.015	0.017
Bond angles (°)	1.89	1.69	1.78

^a^Diffraction data from one crystal was merged into a complete dataset.

^b^Highest resolution shell is shown in parenthesis.

r.m.s.d., root-mean-square deviation.

The overall structure of HLA-A*11:01 in complex with various peptides (SSCSSCPLSK, SSCPLSK or SSCSSC + SSCPLSK) is similar to the standard MHC class I molecules^27^. When overall Cα atoms of the HLA heavy chains from the three structures were superimposed, they showed a root mean square deviation (r.m.s.d.) of 0.3–0.45 Å (Supplementary Fig. 7). Continuous and unambiguous electron density was observed for both SSCSSCPLSK (10-mer) and SSCPLSK (7-mer) in the antigen-binding cleft of HLA-A*11:01 (Fig. 6a–d). For HLA-A*11:01-SSCSSC + SSCPLSK complex, the two short peptides were clearly seen as separate entities that were ordered and displayed continuous electron density (Fig. 6e,f). However, insufficient electron density for the residues SSC in SSCPLSK peptide of the combitope did not allow for precise definition. This observation is not unusual as several structural studies had previously demonstrated that long non-canonical lengths of peptides binding to HLA class I alleles displayed similar poorly defined electron density in the super bulged central region with high flexibility, e.g. a 16-mer self-peptide binding to HLA-B*41:03^28^ and a 15-mer self-peptide binding to HLA-A*02:01^29^. These examples suggest that the ‘SSC’ portion of the C-terminal peptide is highly flexible and mobile in the 5GRG structure.

Overlaying the Cα-atoms of 7-mer (SSCPLSK) with 10-mer (SSCSSCPLSK) peptide revealed a r.m.s.d. of 2.16 Å (Fig. 6g). The 10-mer peptide adopts a 3.4 Å bulged conformation upwards compared to its 7-mer counterpart; as P4-Ser and P5-Ser of the 10-mer orientate towards the exterior, while residues P4-Ser and P5-Ser of the 7-mer are seated in the cleft and thus adopts an extended conformation. Superimposing the Cα-atoms of SSCSSC from the combitope (SSCSSC + SSCPLSK) with 10-mer peptide revealed the orientation of residues to be similar in both structures with a r.m.s.d. of 0.3 Å (Fig. 6g). Overlaying the Cα-atoms of PLSK from the combitope with 10-mer peptide showed a r.m.s.d. of 1.6 Å. The P7-Pro of the combitope is flipped outwards from the antigen-binding cleft by 5.8 Å in comparison to 10-mer peptide, and the side chain of P8-Leu in the combitope is orientated in the opposite direction to the 10-mer peptide (Fig. 6g).

### Interactions with the antigen-binding cleft

**Figure 7:**
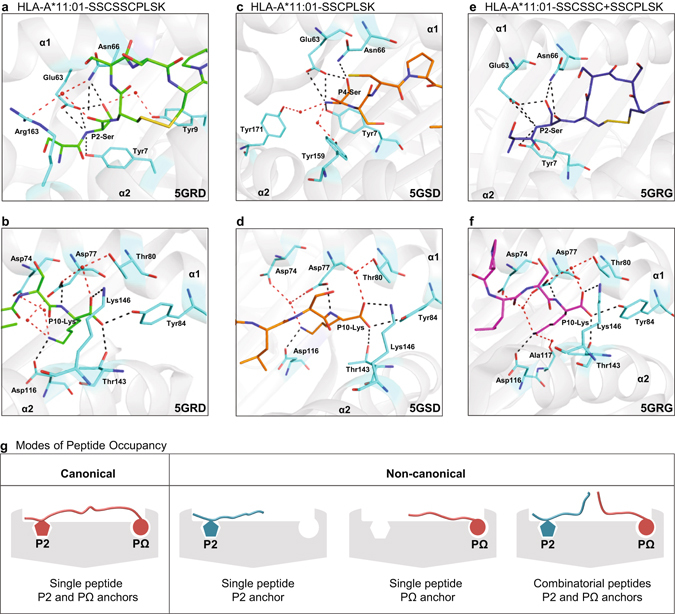
Interactions between primary anchor residues at the N- and C-termini of the peptides and the cleft of HLA-A*11:01. (**a**) Interactions of P2-Ser of SSCSSCPLSK (green) with HLA-A*11:01. (**b**) Interactions of P10-Lys of SSCSSCPLSK (green) with HLA-A*11:01. (**c**) Interactions between N-terminal residue P4-Ser of SSCPLSK (orange) and heavy chain (**d**) Interactions between P10-Lys of SSCPLSK (orange) and HLA-A*11:01 heavy chain. (**e**) Interactions of primary anchor residue P2-Ser of SSCSSC (from SSCSSC + SSCPLSK) (dark blue) when bound to HLA-A*11:01. (**f**) P10-Lys of SSCPLSK (from SSCSSC + SSCPLSK) (magenta) when bound to HLA-A*11:01. Peptide residues are shown in sticks following the same colour scheme. The interacting residues of HLA-A*11:01 molecule are shown in cyan sticks. The direct hydrogen bonding and salt bridges between peptide residues and HLA molecule are shown as black dashed lines. The selected water molecules are shown as red spheres and the interactions mediated via water molecules are shown as red dashed lines. (**g**) Summary diagram indicating the canonical and non-canonical modes of peptide occupancy in MHC-I for antigen presentation.

The 10-mer peptide is anchored into the binding cleft predominantly by residues P2-Ser and P10-Lys making polar and non-polar interactions with HLA-A*11:01 (Supplementary Table 2). The B pocket of HLA-A*11:01 is lined with polymorphic residues suited to accommodate small aliphatic amino acids such as serine^30^. The side chain of P2-Ser is hydrogen bonded to Glu63 and Asn66, whereas its main chain carbonyl makes hydrogen bond with Asn66. Apart from interacting with Tyr7 and Glu63 with its main chain amino group, P2-Ser also interacts with Tyr9, Asn66 and Arg163 via water-mediated interactions (Fig. 7a). Furthermore, the F pocket of HLA-A*11:01 possesses a strong negatively charged environment suited to fit positively charged residues lysine or arginine^30^. This is clear from various interactions between P10-Lys and negatively charged residues Asp74, Asp77 and Asp116 of the F pocket. Additionally, the main chain amino group of P10-Lys interacts with Asp77, and its carboxylate forms a number of direct hydrogen bonds to residues Tyr84, Thr143 and Lys146 of the antigen-binding cleft. The carboxylate of P10-Lys also makes interactions with Asp77 and Thr80 via water molecule (Fig. 7b).

Despite having an unoccupied A pocket in the antigen-binding cleft, SSCPSLK is held with the main chain amino group of P4-Ser hydrogen bonded to Tyr7 and Glu63, and interacts with Tyr159 and Tyr171 via two water molecules (Supplementary Table 3). Moreover, the side chain of P4-Ser makes a hydrogen bond each with Glu63 and Asn66 (Fig. 7c). At the C-terminal of the 7-mer peptide, P10-Lys anchor residue is bound to various residues of F pocket in a similar manner as that in 10-mer peptide (Fig. 7d).

Close examination of the combitope (SSCSSC + SSCPLSK) revealed that the N-terminal peptide, SSCSSC, has its anchor residue P2-Ser seated in B pocket of antigen-binding cleft and is held by various hydrogen and van der Waals interactions (Supplementary Table 4). As shown in Fig. 7e, P2-Ser interacts with residues Tyr7, Glu63 and Asn66 in a fashion similar to 10-mer peptide (Fig. 7a) but without water-mediated interactions. As for the C-terminal peptide (SSCPLSK) of the combitope, it is held firmly in the antigen-binding cleft by primary anchor residue P10-Lys that makes interactions with Asp74, Asp77, Thr80, Tyr84, Asp116, Thr143 and Lys146 in a similar manner to the 10-mer peptide (Fig. 7f, Supplementary Table 4). However, the side chain of P10-Lys in the combitope makes an additional water-mediated interaction with residue Ala117 (Fig. 7f) which likely contributed to the overall stability of the complex. Taken together, the results provide strong evidence that peptides <8-mer can form effective bonding to reside within the peptide binding cleft and provide overall stability to form pHLA complexes.

## Discussion

Following the demonstration of an enrichment in optimal peptide lengths of 8- to 10-mer as well as the presence of certain preferred anchor residues from the peptide elution of MHC-I molecules in 1990^2^, CD8 epitope discovery in relation to MHC-I largely emphasizes on peptides that fall within this range of peptide lengths. Though the majority of the MHC-I presented peptides are of 8- to 10-mer, it remains elusive if peptides shorter than this optimal range possessing only a single anchor residue could stabilize pHLA complexes and evoke an antigen-specific CD8^+^ T cell response as standard proteasome produces proteolytic products that are mostly shorter than the optimal peptide length^10, 11^.

Among the EBV antigens expressed during its life cycle, LMP2 elicits strong CTL responses from EBV carriers in the latency phase^31^. In this study we selected a well-characterized T cell epitope from the EBV LMP2_(340–349)_ peptide^13–15^ to investigate the heterogeneity of peptides that are capable of eliciting T cell responses. Using progressively truncated peptides starting from the canonical SSCSSCPLSK 10-mer peptide, we showed that truncated versions of the 10-mer were able to stimulate specific CD8^+^ T cell responses from PBMCs of healthy HLA-A*11 positive donors previously exposed to EBV. The intensity of CD8^+^ responses to these single truncated peptides were generally lower than the response to the full-length peptide but demonstrated that peptides <8-mer in length are not entirely non-functional.

Although progressively truncated LMP2_(340–349)_ of 2- to 5-mer length no longer bound HLA in a stable manner as a single peptide, they were able to complement and form a stable pHLA complex with two peptides concurrently occupying the antigen binding groove (Fig. 4a). These exogenously supplied peptides *in vitro* are likely to enter the endoplasmic reticulum (ER) via a variety of pathways that include TAP-independent pathways and autophagy^32, 33^. Under physiological conditions however, peptides that are <8-mers are abundantly generated by proteasomes^10^ and can be transported into the ER via classical TAP pathway^34^. Long peptides in the ER can also generate shorter peptide fragments due to protease activity or trimming by ERAP^35, 36^. The use of minigenes to express truncated peptides intracellularly provided clear evidence that these peptides can assemble with HLA similar to the exogenously supplied peptides and present it on the cell surface to stimulate a CD8^+^ T cell response. Our data suggest that the availability of short peptide fragments can generate stable pHLA complexes and over time can stimulate CD8^+^ responses to combinatorial mixtures of these peptide fragments. As the binding of the truncated peptides is lower than that of the full-length peptide, the stimulation of T cells in the body probably takes place over a longer period of time. However, with EBV being a persistent latent infection in most affected individuals, the constant supply of viral latent phase proteins are likely to provide regular antigenic challenge for re-stimulation.

The resolved crystal structures in this study have also shed light on how a truncated short peptide with a single anchor residue as well as a combination of two of such peptides, are able to sit securely within the HLA antigen-binding cleft. Though it had been previously demonstrated in mouse H-2D^b^ to bind a 5-mer peptide (NYPAL)^37^, this could be accounted for by H-2D^b^ having two anchor pockets at P5 and P9^38^ and thereby justifying for the stabilization of the 5-mer at the two anchor residues (i.e. NYPAL). This is in contrast to our results that revealed the ability of peptide <8-mer with a single anchor residue binding singly or cooperatively with a second peptide to the HLA antigen-binding cleft. Furthermore, our crystal structure for the dual peptide combination revealed a ‘bulged’ conformation, similar to that initially solved bulged 13-mer peptide structure in rat RT1-A^a^ in 2001^39^ as well as the various HLA class I structures with highly flexible binding long peptides^7, 28, 29^.

Moreover, the resolved crystal structures have also provided an explanation for the diversity in TCRVβ usage by the T cells that recognized the different peptide lengths and combinations. In the structure formed by the C-terminal 7-mer peptide (5GSD), no intra-peptide disulfide bond was formed as it contains a single cysteine; while in the structure containing the dual short peptide combination (SSCSSC + SSCPLSK; PBD ID: 5GRG), the N-terminal peptide (SSCSSC) forms an intra-peptide disulfide bond with the C-terminal peptide (SSCPLSK) being highly flexible and in particularly with P7-Pro exposed. These structures offers insights into the altered surface contours of the peptide-HLA complex facing the T cell receptor, whereby the two complementary peptides SSCSSC + SSCPLSK forms a distinctly altered surface contour presented to the T cell receptor compared to the native 10-mer and the C-terminal 7-mer peptides. Thus it is likely that the neo-epitopes presented by complementary peptides will stimulate a wide diversity of T cell receptors since the subtle changes created by short C-terminal peptides has resulted in an increase in TCRβ chain usage (Fig. 2).

This is the first demonstration of a pHLA tetra-complex structure that contains two non-contiguous peptides. Our data thus provides a fundamental new approach to thinking about peptide occupancy in the human MHC-I molecule (Fig. 7g), which has implications for many aspects, including CD8 epitope discovery and vaccine development. Additionally, the sources of these short peptides of <8-mer can be from self or non-self antigens, thereby providing an immense potential for generating neoepitopes. This may be relevant to how we think about molecular mimicry^40^ and its role in the development of diseases such as autoimmunity. With the possibility that a combination epitope can be formed by part self-antigen and part non-self antigen, this greatly increases the chance that T cells triggered by a pathogen may cross-react with self-antigens. Such auto-reactive T cells may originate initially in very low numbers and of low-affinity and expand in numbers over time with repeated re-stimulation. Recent studies have demonstrated that peptide presentation by MHC can be of non-contiguous origin in both MHC class I^41^ and class II^42^, suggesting an added level of complexity to the generation of T cell epitopes. While the peptides described in these studies have a relatively restricted range of peptide fusion partners, our data reveals the immense potential of mix-and-match peptides for the generation of T cells neoepitopes.

In addition, future work is required to examine if auto-reactive CD8 T cells that recognize such combination neoepitopes are enriched in patient cohorts with specific autoimmune diseases. Nonetheless, our results demonstrated with the use of PBMCs from a healthy subject reveal the prospect of this mechanism. The identification of novel forms of neoepitopes will greatly enhance the understanding of diseases that involve immune recognition of self antigens such as autoimmunity and cancer; and also in infectious diseases in which the diversity of T cell response to non-canonical short peptides form additional layers of immune response that has not been well understood before.

## Materials and Methods

### HLA-typing of donors and *in-vitro* stimulation of human PBMCs

With approval from the IRB of the National University of Singapore, peripheral blood was obtained from healthy donors following written informed consent and all experiments were performed in accordance to relevant guidelines and regulations. HLA of donors were identified using sequence based typing as described previously^43^. PBMCs were isolated with Ficoll-Paque PLUS (GE Healthcare) and maintained in RPMI 1640 supplemented with 5% human AB serum (Sigma), 100 IU/ml penicillin-streptomycin (Gibco), 2 mM L-glutamine (Sigma) and 10 mM HEPES (Sigma). For 14-day cultures, PBMCs (1 × 10^6^ cells/ml) were incubated with 10 µM peptides (GenScript) and supplemented with 25 U/ml rIL2 (R&D Systems). Culture medium with rIL2 was replenished every 2–3 days from Day 5 onwards.

### IFN-γ ELISpot assays

IFN-γ secretion was detected using Human IFN-γ ELISpot Kit according to manufacturer’s instructions (Mabtech). Briefly, PBMCs resuspended in RPMI 1640 with 10% fetal calf serum, 100 IU/ml penicillin-streptomycin, 2 mM L-glutamine and 10 mM HEPES were seeded in duplicate wells into a 96-well PVDF plate pre-coated with anti-IFN-γ mAb (1-D1K), at 5 × 10^5^ cells per well in the presence of 10 µM peptide. HLA-A*24-restricted TYGPVFMCL (EBV LMP2_419–427_) served as the control peptide in donors without HLA-A*24, while HLA-A*02-restricted YLLEMLWRL (EBV LMP1_125–133_) served as the control in those with the allele. Following 2-day incubation, plates were washed and incubated with alkaline phosphatase (ALP)-conjugated anti-IFN-γ mAb (7-B6-1) for 2 h. Plates were washed again prior to colour development with ALP substrate NBT. Wells were scanned and counted with CTL ImmunoSpot® S6 FluoroSpot Analyzer (Cellular Technology Limited).

### Expression, refolding and native western blot assessment of pHLA complexes

Expression and refolding of the pHLA complexes were carried out as previously described^44, 45^. Briefly, recombinant human HLA class I allele (exon 2–4) and human β2 m were expressed and solubilized in 8 M urea. Stock peptides (Genscript, USA) at 10 mg/ml DMSO were added to HLA and β2 m in refolding buffer for 72 h, followed by dialysis against 10 mM Tris-HCl (pH 8.0) at 4 °C overnight. Thereafter, the mixtures were concentrated using Vivaspin (Sartorius). To assess the stability of the pHLA complexes, the samples were separated on native polyacrylamide gel electrophoresis and transferred onto PVDF membranes (GE Healthcare). Western blot analyses were then carried out with W6/32 mouse mAb (DAKO) as the primary antibody, followed by HRP-conjugated goat anti-mouse secondary antibody (DAKO). Immunoreactive bands indicative of stable pHLA were then detected using ECL Plus western blotting substrate (Pierce).

### Preparation of pHLA tetramers

HLA-peptide tetramers were produced as previously described^46^. Briefly, to form stable pHLA complexes, peptides SSCSSCPLSK (EBV LMP2_340–349_), SSSPQCPLSK (hybrid of human p53_313–317_ and EBV LMP2_345–349_), KICMQCPLSK (hybrid of human ribosomal protein S4_63–67_ and EBV LMP2_345–349_) and KTYGECPLSK (hybrid of human NADH dehydrogenase subunit C2_106–110_ and EBV LMP2_345–349_) were refolded individually with HLA heavy chain and β2 m in refolding buffer for 72 h. Refolded products were then dialyzed against 10 mM Tris-HCl (pH 8.0) at 4 °C overnight. Dialyzed complexes were purified by anion exchange chromatography using HiPrep DEAE 16/10 column (GE Healthcare) equilibrated with 10 mM Tris-HCl (pH 8.0), followed by gel filtration with a HiLoad 16/60 Superdex 75 preparatory-grade GF column (GE Healthcare). The purified, refolded pHLA monomeric complexes were then biotinylated by recombinant BirA enzymes. Tetrameric pHLA complexes were assembled by the stepwise addition of streptavidin-phycoerythrin (PE) (Invitrogen) or streptavidin-allophycocyanin (APC) (BioLegend) at a molar ratio of 4:1.

### Tetramer staining

Cells from the 14-day cultures were harvested, washed with PBS and stained with 12 µg/ml PE-conjugated pHLA tetramer for 20 min, followed by incubation with BV421-conjugated anti-human CD8 (BD Biosciences) for 15 min. Cells were washed with PBS and analysed with LSR II flow cytometer (BD Biosciences). An aliquot of cells from the 14-day cultures was stained with propidium iodide to determine viability and gating parameters. These gating values were utilised for cells stained with PE-conjugated tetramers and BV421-conjugated anti-human CD8 antibodies. Data analyses were performed using FlowJo (Tree Star Incorporated). TYGPVFMCL and YLLEMLWRL peptides served as controls.

### Intracellular cytokine staining

14 days post-peptide stimulation, PBMC cultures were resuspended in fresh culture medium at 1 × 10^6^ cells/ml. Cells were restimulated with 10 µM of their respective peptides for 5 h together with BD GolgiPlug^TM^ protein transport inhibitor (BD Biosciences) and fluorescein isothiocyanate (FITC)-conjugated anti-CD107a antibody (BD Biosciences). TYGPVFMCL peptide served as the control. Following stimulation, cells were stained with PE-conjugated pHLA-tetramer and BV421-conjugated anti- human CD8 prior to fixation and permeabilization with Cytofix/ Cytoperm solution (BD Biosciences). Intracellular cytokine staining was performed with APC-conjugated anti-IFN-γ (BD Biosciences) before washing with PBS and subsequent flow cytometry analysis.

### Construction and electroporation of minigene plasmids

Construction and electroporation of minigenes plasmids were performed as previously described^47, 48^ with modifications. Minigene expression plasmids were constructed by cloning DNA inserts encoding for EBV LMP2_340–349_ (SSCSSCPLSK), EBV LMP2_340–345_ (SSCSSC) and EBV LMP2_343–349_ (SSCPLSK) into pcDNA3.1 + vector (Invitrogen) using *NheI* and *XhoI* restriction sites. The DNA inserts were generated by annealing their corresponding complementary DNA sequences with flanking *NheI* and *XhoI* restriction sites (LMP2-340-349-F: 5′-CTAGCATGTCTTCGTGCTCTTCATGTCCACTGAGCAAGTAATAAC-3′, LMP2-340-349-R: 5′-TCGAGTTATTACTTGCTCAGTGGACATGAAGAGCACGAAGACATG-3′; LMP2-340-345-F: 5′-CTAGCATGTCTTCGTGCTCTTCATGTTAATAAC-3′, LMP2-340-345-R: 5′-TCGAGTTATTAACATGAAGAGCACGAAGACATG-3′; LMP2-343-349-F: 5′-CTAGCATGTCTTCATGTCCACTGAGCAAGTAATAAC-3′, LMP2-343-349-R: 5′-TCGAGTTATTACTTGCTCAGTGGACATGAAGACATG-3′ (AITbiotech, Singapore)). Minigene constructs were electroporated into donor’s PBMC using Amaxa® Human B cell Nucleofactor® Kit (Lonza) according to manufacturer’s instruction. Electroporated cells were washed with PBS 6 h later and co-incubated with non-electroporated PBMCs in culture medium supplemented with 25 U/ml rIL2 at 37 °C with 5% CO_2_ for 14 days and replenished every 2–3 days with rIL2 medium from Day 5 onwards.

### TCRVβ characterization

TCRVβ characterization was performed as previously described^49^ with modifications. Briefly, PBMC from each unique anonymized HLA-A*11:01 positive donor was split into three samples and individually stimulated with 10 µM of specified peptides at 2 × 10^6^ cells/ well. Cells were cultured for 14 days with rIL2-supplemented medium replenished every 2–3 days from Day 5 onwards. Cells harvested for staining were first stained with a panel of antibodies specific for different TCRVβ (IOTest Beta Mark Kit, Beckman Coulter) and then further stained with APC-conjugated A*11:01/SSCSSCPLSK tetramer, followed by flow cytometry analysis.

### Thermal stability assay

To examine the stability of the various HLA-A*11:01-peptide complexes, a thermal shift assay was performed as described previously^49, 50^ with SYPRO Orange fluorescent dye (Molecular Probes) on LightCycler II 480 real time PCR system (Roche). Each refolded pHLA complex or control reaction in 10 mM Tris buffer pH 8 was set in duplicates together with SYPRO Orange dye and heated from 20 °C–95 °C with continuous ramp rate of 0.04 °C/s with 15 acquisitions/°C. Fluorescence intensity was measured with excitation wavelength 490 nm and emission wavelength 575 nm. The raw data was normalized, processed and plotted as negative first derivatives; where a sharp single negative peak corresponds to the respective Tm.

### Crystallization and data collection

HLA-A*11:01-SSCSSCPLSK, HLA-A*11:01-SSCPLSK and HLA-A*11:01-SSCSSC + SSCPLSK complexes were concentrated to 10 mg/ml in 10 mM Tris-HCl (pH 8.0). Crystals for all the three peptide-HLA complexes were obtained by hanging–drop vapour-diffusion method at 16 °C. The crystallization solution for HLA-A*11:01-SSCSSCPLSK comprised of 0.2 M ammonium acetate, 0.1 M sodium acetate trihydrate (pH 4.6) and 30% PEG 4000; HLA-A*11:01-SSCPLSK consisted of 0.2 M ammonium acetate and 20% PEG 4000; HLA-A*11:01-SSCSSC + SSCPLSK comprised of 0.1 M ammonium acetate, 0.1 M Bis-Tris (pH 5.5) and 22% PEG 4000. Before data collection, the crystals were equilibrated in respective crystallization solutions with 15% glycerol added as a cryoprotectant, and then flash frozen in liquid nitrogen. For HLA-A*11:01-SSCSSCPLSK and HLA-A*11:01-SSCSSC + SSCPLSK, data were recorded on an ADSC Quantum 315r CCD detector at beamline BL13B1 at the National Synchrotron Radiation Research Centre (Taiwan) at wavelength 0.999 Å. These two datasets were indexed, integrated and scaled using HKL2000 suite of programs. For HLA-A*11:01-SSCPLSK, a single wavelength native data (1.54178 Å) was collected with the CCD Proteum X-ray diffraction system (Institute of Molecular and Cell Biology, A*STAR, Singapore), and the datasets were indexed, integrated and scaled by Proteum2. All the three datasets were collected at a temperature of 100 K. Details of the data processing statistics are given in Table 1.

### Structure determination and refinement

The structure of all the three HLA-A*11:01-peptide complexes in this study were determined by molecular replacement using the coordinates from the structure of HLA-A*11:01^51^ with the peptide removed (PDB accession code 1X7Q). Molecular replacement was performed using the PHASER program^52^ for HLA-A*11:01-SSCSSCPLSK and HLA-A*11:01-SSCSSC + SSCPLSK, whereas AUTO-MR^53^ was used for HLA-A*11:01-SSCPLSK. Manual model building was conducted using Coot software followed by refinement with REFMAC5. The final models were validated using PROCHECK^54^ with all dihedral angles in the favoured or allowed regions. Structural analysis indicated that 97.59%, 96.70% and 97.32% of residues were in favoured Ramachandran regions and 2.41%, 3.24% and 2.68% were in allowed regions for HLA-A*11:01-SSCSSCPLSK, HLA-A*11:01-SSCPLSK and HLA-A*11:01-SSCSSC + SSCPLSK respectively. The final refinement statistics for all structures are summarized in Table 1. The atomic coordinates and structure factors have been deposited in the PDB database as HLA-A*11:01-SSCSSCPLSK (PDB code 5GRD), HLA-A*11:01-SSCPLSK (PDB code 5GSD) and HLA-A*11:01-SSCSSC + SSCPLSK (PDB code 5GRG). All molecular graphics representations were generated using PyMol^55^. The interactions between the peptides and the HLA have been calculated using CONTACT in the CCP4 software suite.

## Electronic supplementary material

Supplementary Figures and Tables
