# Supplementary material for: Dual non-contiguous peptide occupancy of HLA class I evoke antiviral human CD8 T cell response and form neo-epitopes with self-antigens
Source: Sci Rep. 2017 Jul 11;7:5072. doi: 10.1038/s41598-017-05171-w (PMC5505988; doi:10.1038/s41598-017-05171-w)
Supplement: Supplementary file 1 — Supplementary Figures and Tables [file 41598_2017_5171_MOESM1_ESM.pdf]

Supplementary Figures and Tables

Title: Dual non-contiguous peptide occupancy of HLA class I evoke antiviral human CD8 T cell response and form neo-epitopes with self-antigens

Authors: Ziwei Xiao, Zhiyong Ye, Vikeramjeet Singh Tadwal, Meixin Shen and Ee Chee Ren

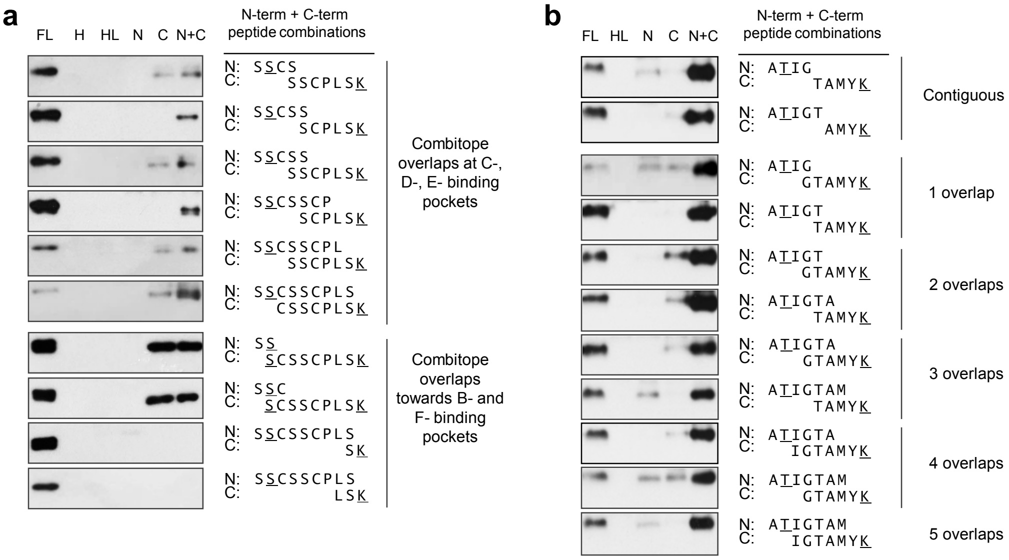

Supplementary Figure 1. pHLA complexes are stabilized by the binding of two truncated peptides (**a**) *In-vitro* refolding of HLA-A*11:01 and β_2m with combined epitopes (combitopes) comprising of short EBV LMP2 N-terminal and C-terminal peptides (N+C) overlapping at C-, D- and E- binding pockets of the MHC binding cleft formed stable pHLA complexes. Minimal or a lack of stabilized pHLA complexes was observed with individual N-terminal (N) or C-terminal (C) short peptides. Overlaps of amino acid residues of short peptides towards the B- and F-binding pockets failed to enhance pHLA complex stabilization. Refolding of HLA-A*11:01 heavy chain, β_2m light chain and SSCSSCPLSK peptide served as the positive control (FL), while heavy chain (H), heavy and β_2m light chain (HL) served as the negative controls. (**b**) Contiguous and overlapping combitopes (N+C) from another HLA-A*11-restricted peptide, ATIGTAMYK (EBV BRLF1$_{134\text{-}142}$) were also able to stabilize pHLA complexes. Refolding of HLA-A*11:01 heavy chain, β_2m light chain and 9-mer ATIGTAMYK served as the positive control (FL), while heavy and β_2m light chain (HL) served as the negative controls. 'N' and 'C' indicate N-terminal (N-term) and C-terminal (C-term) peptides respectively.

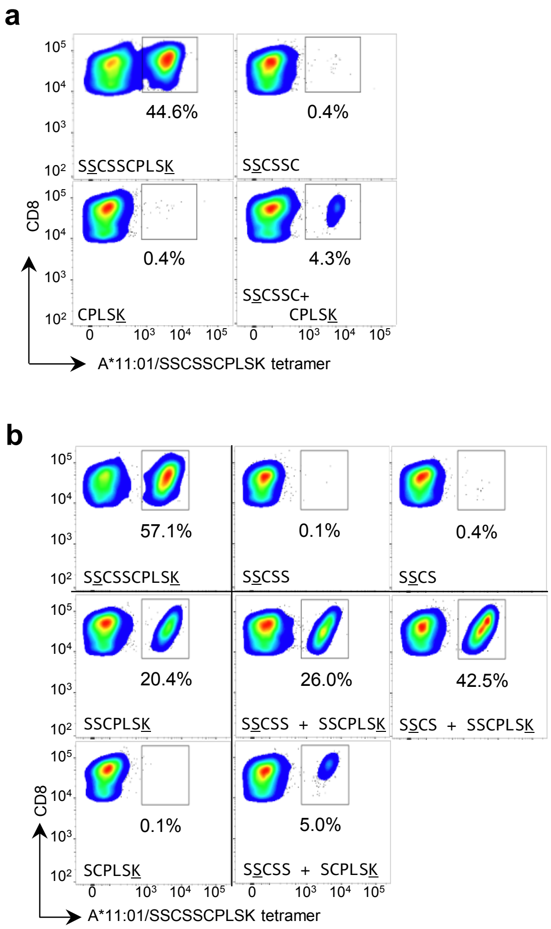

Supplementary Figure 2. Overlapping combitopes recall CD8 and A*11:01/SSCSSCPLSK tetramer double-positive T cells

Several other overlapping combitopes as shown in (**a**) (SSCSSC+CPLSK) and (**b**) (SSCS+SSCPLSK, SSCSS+SCPLSK and SSCSS+SSCPSLK) were able to activate CD8 and A*11:01/SSCSSCPLSK tetramer double-positive T cells in a synergistic manner in 14-day PBMC cultures of a representative HLA-A*11:01 homozygous donor with the indicated peptides.

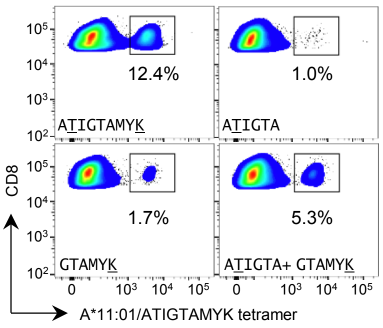

Supplementary Figure 3. Overlapping combitopes recall CD8 and A*11:01/ATIGTAMYK tetramer double-positive T cells

Overlapping combitope (ATIGTA+GTAMYK) from another A*11-restricted ATIGTAMYK peptide was also able to activate CD8 and A*11:01/ATIGTAMYK tetramer double-positive T cells in a synergistic manner in a 14-day PBMC culture of a representative HLA-A*11:01 donor.

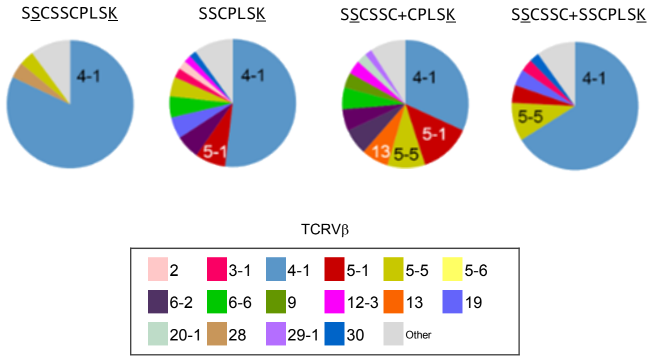

Supplementary Figure 4. TCRVβ usage of T cells stimulated by combinatorial short peptides
TCRVβ usage in A*11:01/SSCSSCPLSK tetramer positive T cells stimulated with the indicated peptides in 14-day culture of PBMCs from a representative HLA-A*11:01 homozygous individual.

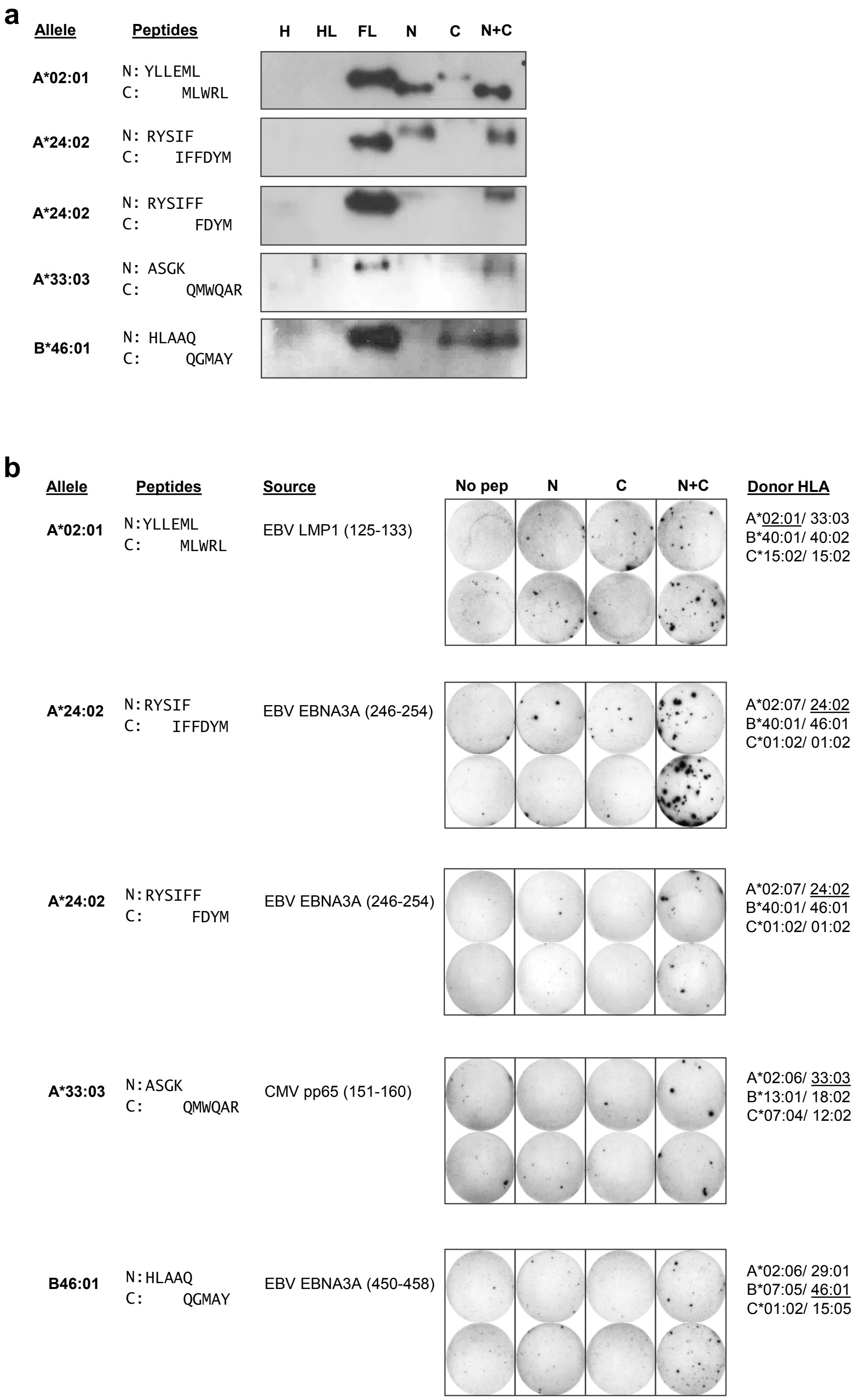

Supplementary Figure 5. Combinations of truncated peptides can stabilize various pHLA complexes and induce CD8 T cell recall response as evaluated by IFN-γ ELISpot

(**a**) *In-vitro* refolding of HLA-A*02:01, A*24:02, A*33:03 and B*46:01 heavy chains and β_2m with truncated peptides comprising different N-terminal and C-terminal viral peptides (N+C) formed stable pHLA complexes. Individual truncated N-terminal (N) and C-terminal (C) peptides alone had little or no stabilization effect on the pHLA complexes. Refolding of heavy chain alone (H), heavy chain and β2m (HL) served as negative controls, while refolding of heavy chain, β2m with the full-length peptide (FL) served as the positive control. (**b**) PBMCs from various donors (with HLA allele of interest underlined) were stimulated with the indicated N-terminal (N), C-terminal (C) truncated peptides or a combination of both (N+C) and evaluated by IFN-γ ELISpot. PBMCs without any peptide stimulation (No pep) served as negative control. EBV, Epstein-Barr Virus; CMV, Cytomegalovirus.

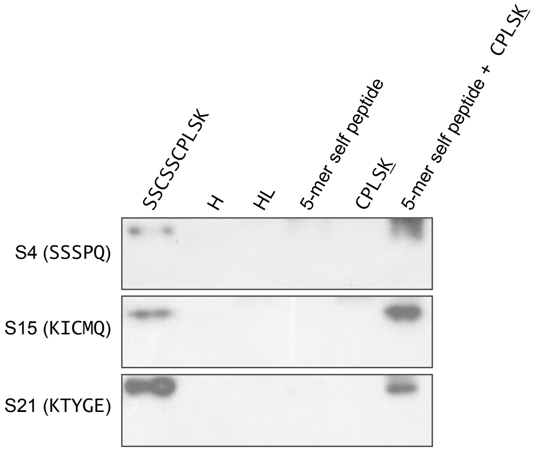

Supplementary Figure 6. *In-vitro* refolding of self and non-self combinatorial peptides
Enhanced stabilized pHLA complexes observed in in-vitro refolding with combined epitopes (combitopes) comprising of EBV LMP2 C-terminal 5-mer and Self-N5 peptide (5-mer self peptide + CPLSK) for the various self peptides identified in Fig. 5b, namely S4 (SSSPQ from p53), S15 (KICMQ from ribosomal protein S4) and S21 (KTYGE from NADH dehydrogenase subunit C2). Minimal or no stabilized pHLA complexes are observed in refolding with self N-terminal 5-mer (5-mer self peptide) or EBV LMP2 C-terminal 5-mer peptide (CPLSK). Refolding with SSCSSCPLSK served as positive controls. HLA-A*11:01 heavy chain (H), heavy chain and β_2M light chain (HL) served as negative controls.

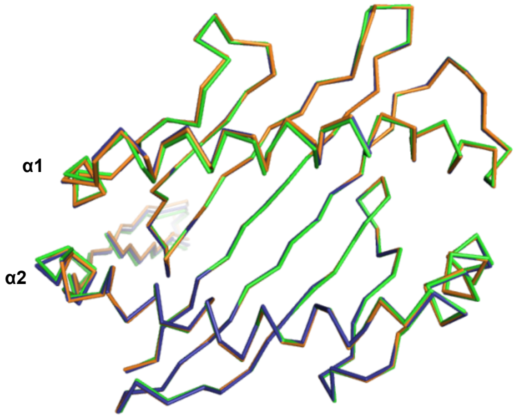

(PDB Code: 5GRD) HLA-A*11:01-SSCSSCPLSK
(PDB Code: 5GSD) HLA-A*11:01-SSCPLSK
(PDB Code: 5GRG) HLA-A*11:01-SSCSSC+SSCPLSK

Supplementary Figure 7. Overlay of HLA-A*11:01 heavy chain helices from the three HLA-A*11:01-epitope crystal structures.

Superposition of α1 and α2 domains of HLA-A*11:01 when SSCSSCPLSK (green), SSCPLSK (orange) and SSCSSC+SSCPLSK (dark blue) are bound; showing the overall similarity (r.m.s.d. 0.3-0.45 Å) between the three HLA-A*11:01 heavy chains. The α1 and α2 domains are represented in ribbon and the peptides were excluded from the diagram.

Supplementary Table 1. TCRVβ repertoire of T cells activated by full-length, C-terminal 9-mer and 8-mer short peptides in eight donors.

	SSCSSCPLSK								SCSSCPLSK								CSSCPLSK							
TCRVβ \ Donor	1	2	3	4	5	6	7	8	1	2	3	4	5	6	7	8	1	2	3	4	5	6	7	8
2	0.0	1.5	4.3	8.5	2.4	1.7	0.5	0.7	0.4	1.8	3.7	7.8	2.6	6.4	0.0	1.8	0.7	1.3	**12.4**	8.5	5.5	9.4	**17.6**	5.4
3-1	0.1	1.1	0.0	2.3	1.1	0.0	0.1	1.1	0.1	0.5	0.0	1.0	1.9	0.0	0.0	0.7	0.1	0.5	0.0	1.6	2.5	0.0	7.1	3.5
4-1	**97.8**	**93.5**	**66.1**	**54.4**	**27.6**	**22.8**	**89.6**	0.6	**90.4**	**95.0**	**64.3**	**54.7**	9.9	**81.9**	**45.6**	2.0	**94.8**	**92.7**	**79.1**	**72.1**	**12.9**	**88.5**	**13.6**	4.3
4-3	0.0	0.0	0.1	0.2	0.0	0.0	0.0	0.5	0.0	0.0	0.0	0.1	0.0	0.0	1.3	0.6	0.1	0.0	0.0	0.2	0.0	0.0	3.6	3.0
5-1	0.1	2.3	7.6	**23.9**	7.7	3.1	3.7	1.9	2.2	1.7	7.0	**21.5**	**14.8**	6.6	2.6	3.9	8.3	3.5	3.2	**17.8**	**17.4**	6.7	**15.2**	8.8
5-5	0.7	1.2	0.0	0.0	0.5	0.0	3.9	0.3	7.2	1.7	0.0	0.0	0.7	0.0	**13.6**	2.2	2.7	2.5	0.0	0.0	1.2	0.0	**10.2**	2.1
5-6	0.0	1.9	0.9	9.0	0.7	2.7	2.2	0.6	0.4	2.7	1.2	6.7	0.5	8.4	1.1	1.2	0.5	1.5	4.4	9.2	0.6	**11.5**	0.0	4.1
6-2	0.2	2.0	7.3	9.8	1.5	0.0	1.1	1.2	0.8	2.3	5.8	**12.1**	2.4	8.9	2.5	0.8	3.4	1.6	3.3	7.5	2.1	7.5	1.8	5.1
6-5	0.3	0.4	1.2	3.6	1.1	0.0	0.3	0.5	0.4	0.7	1.6	4.2	1.4	0.0	0.0	1.1	1.6	0.7	5.6	3.9	2.6	0.0	0.0	0.5
6-6	0.3	3.6	2.2	**15.6**	3.0	1.5	2.4	3.9	0.2	2.9	3.5	**12.0**	1.3	9.8	1.5	1.1	6.4	1.0	3.6	**13.2**	1.8	9.6	0.0	4.5
9	0.3	1.1	5.9	5.4	3.6	3.4	5.5	4.2	3.1	0.4	3.0	8.9	3.3	0.8	0.0	**25.4**	5.2	2.2	4.6	2.8	6.3	6.7	9.8	**15.8**
10-3	0.0	0.1	0.8	0.0	1.1	0.0	0.0	0.9	0.1	0.3	0.4	0.5	2.3	0.0	0.0	0.7	0.0	0.4	0.8	0.4	4.8	0.0	0.0	1.0
11-2	0.5	0.1	0.1	0.2	0.4	0.0	1.1	2.4	0.3	0.1	0.0	0.8	0.6	0.0	0.0	1.2	1.7	0.1	0.4	0.7	3.1	0.0	0.0	2.0
12-3	0.1	1.8	2.5	9.1	2.9	0.0	4.3	**84.5**	0.1	1.3	2.5	4.6	2.9	0.0	0.0	**63.7**	0.8	2.2	2.8	3.9	4.6	0.0	0.0	**14.3**
13	0.2	1.3	**16.3**	9.3	4.5	0.0	0.9	1.8	0.1	3.6	**13.9**	9.5	5.3	0.0	1.2	**23.1**	1.3	1.6	5.4	3.7	5.8	0.0	**13.0**	**15.8**
14	0.4	1.0	0.0	1.9	0.2	0.0	0.0	0.4	0.9	1.5	0.0	2.5	0.6	0.0	0.0	0.7	0.1	1.9	0.0	3.7	1.0	0.0	2.7	1.7
18	0.0	0.7	0.6	2.5	5.5	0.0	1.6	0.2	0.4	1.3	0.8	3.3	**21.4**	0.0	1.3	0.3	0.2	0.9	0.0	2.3	8.4	0.0	1.5	0.7
19	0.2	1.9	**12.4**	8.5	2.6	1.9	1.2	1.0	1.6	0.5	**10.1**	2.2	2.2	3.8	1.4	2.6	0.0	2.3	7.2	9.5	3.9	2.1	**28.6**	1.4
20-1	0.0	0.9	2.6	3.6	4.7	0	1.6	1.6	0.1	0.6	4.0	3.9	8.6	0.0	2.3	2.0	0.2	0.8	2.7	7.1	3.3	0.0	0.0	**10.7**
25-1	0.0	0.0	2.9	0.4	0.8	0.0	0.5	0.1	0.0	0.2	0.8	1.8	0.8	0.0	0.0	0.5	0.0	0.0	0.8	2.0	1.4	0.0	1.4	0.8
27	0.1	0.1	3.4	0.7	3.5	**59.7**	0.5	0.3	0.2	1.0	6.6	1.5	2.5	0.0	0.0	0.0	0.2	0.2	3.4	1.0	5.3	0.0	2.7	0.4
28	0.8	4.7	0.0	0.9	4.9	0.0	1.3	0.3	0.3	2.2	0.0	0.3	3.5	0.0	**24.3**	3.3	0.1	1.8	0.0	0.2	7.5	0.0	8.5	5.0
29-1	0.1	0.2	1.2	5.3	1.4	1.1	1.6	1.2	0.3	0.3	0.5	6.6	6.6	0.0	0.0	0.0	1.2	1.0	0.3	7.7	3.3	0.0	0.0	**10.2**
30	0.1	2.5	**10.2**	4.6	0.2	0.0	0.9	0.7	0.6	2.9	**11.9**	6.2	0.3	0.0	0.0	0.9	1.2	1.3	6.4	7.5	0.7	0.0	3.0	2.6

TCRVβ family usage (expressed as percentages of tetramer positive T cells) for each donor are indicated and those greater than 10.0% are in bold.

Supplementary Table 2. Hydrogen bond and Van der Waals interactions between SSCSSCPLSK and HLA-A*11:01

Peptide		Hydrogen-bond[a]/salt bridge[b] interactions			vdW contact residues[c]
**Residue**	**Atom**	**Residue**	**Atom**	**Distance (Å)**	
P1-Ser	N	Tyr7	OH (HB)	2.92	Met5, Tyr7, Glu63, Tyr159, Arg163, Trp167, Tyr171
		Tyr171	OH (HB)	2.61	
	O	Tyr159	OH (HB)	2.69	
	OG	Glu63	OE2 (HB)	2.72	
		Arg163	NH1 (HB)	2.88	
P2-Ser	N	Glu63	OE1 (HB)	3.27	Tyr7, Tyr9, Glu63, Asn66, Tyr99, Tyr159, Arg163
		Glu63	OE2 (HB)	3.00	
		Tyr7	OH (HB)	3.39	
	O	Asn66	ND2 (HB)	3.27	
	OG	Glu63	OE1 (HB)	2.65	
		Asn66	ND2 (HB)	3.07	
P3-Cys	N	Tyr99	OH (HB)	2.99	Asn66, Tyr99, Tyr159
P4-Ser					Gln62, Asn66
P5-Ser					Gln155
P6-Cys					Gln156
P7-Pro					Gln70, Thr73
P8-Leu					Thr73, Ala150, Ala152, Trp147
P9-Ser	O	Trp147	NE1 (HB)	3.03	Asp77, Lys146, Trp147
P10-Lys	N	Asp77	OD1 (HB)	2.84	Asp77, Thr80, Leu81, Tyr84, Asp116, Tyr123, Thr143, Lys146, Trp147
	NZ	Asp116	OD2 (SB)	2.67	
	OT1	Tyr84	OH (HB)	2.66	
		Thr143	OG1 (HB)	2.70	
	OT2	Lys146	NZ (HB)	2.76	
	O	Trp147	NE1 (HB)	3.03	

[a]The cutoff distance of a hydrogen bond (HB) is 3.4 Å.
[b]The cutoff distance for salt bridge (SB) interactions is 4 Å.
[c]The cutoff distance for van der Waals (vdW) contact is 4.0 Å.
[a, b, c] The distances for various interactions were determined using CONTACT from CCP4i suite and COOT. Residues having any atom within this distance are considered to be contact residues.

Supplementary Table 3. Hydrogen bond and Van der Waals interactions between SSCPLSK and HLA-A*11:01

Peptide		Hydrogen-bond[a]/Salt bridge interactions[b]			vdW contact residues[c]
Residue	Atom	Residue	Atom	Distance (Å)	
P4-Ser	N	Tyr7	OH (HB)	3.25	Tyr7, Tyr9, Glu63, Asn66, Tyr99, Tyr159
		Glu63	OE1 (HB)	3.31	
		Glu63	OE2 (HB)	3.01	
	OG	Asn66	ND2 (HB)	2.92	
		Glu63	OE2 (HB)	2.56	
P5-Ser	N	Tyr99	OH (HB)	3.15	Asn66, Tyr99, Tyr159
P6-Cys					Asn66
P7-Pro					Asn66, Ala69, Gln70, Thr73, Gln155
P8-Leu					Thr73, Arg114, Trp147, Ala152, Gln155, Gln156
P9-Ser	O	Trp147	NE1 (HB)	2.94	Asp77, Trp147
P10-Lys	N	Asp77	OD1 (HB)	2.82	Asp77, Tyr84, Ile95, Asp116, Thr143, Lys146, Trp147
	NZ	Asp116	OD2 (SB)	2.60	
	OT1	Tyr84	OH (HB)	2.89	
		Thr143	OG1 (HB)	2.81	
	OT2	Lys146	NZ (HB)	2.79	

[a]The cutoff distance of a hydrogen bond (HB) is 3.4 Å.

[b]The cutoff distance for salt bridge (SB) interactions is 4 Å.

[c]The cutoff distance for van der Waals (vdW) contact is 4.0 Å.

[a, b, c] The distances for various interactions were determined using CONTACT from CCP4i suite and COOT. Residues having any atom within this distance are considered to be contact residues.

Supplementary Table 4. Hydrogen bond and Van der Waals interactions between SSCSSC and HLA*11:01 as well as PLSK and HLA-A*11:01 in a combitope

Peptide within binding groove	Peptide		Hydrogen-bond[a]/Salt bridge[b] interactions			vdW contact residues[c]
	Residue	Atom	Residue	Atom	Distance (Å)	
SSCSSC (of Combitope)	P1-Ser	N	Tyr7	OH (HB)	2.88	Met5, Tyr7, Glu63, Tyr159, Arg163, Trp167, Tyr171
			Tyr171	OH (HB)	2.65	
		O	Tyr159	OH (HB)	2.60	
		OG	Glu63	OE2 (HB)	2.79	
			Arg163	NH1 (HB)	2.90	
	P2-Ser	N	Tyr7	OH (HB)	3.35	Tyr7, Tyr9, Glu63, Asn66, Tyr99, Tyr159, Arg163
			Glu63	OE1 (HB)	3.01	
			Glu63	OE2 (HB)	3.38	
		O	Asn66	ND2 (HB)	3.39	
		OG	Glu63	OE1 (HB)	3.01	
			Asn66	ND2 (HB)	3.01	
	P3-Cys	N	Tyr99	OH (HB)	3.16	Asn66, Tyr99, Tyr159
	P4-Ser					Asn66
	P5-Ser					Gln155
	P6-Cys					Gln70, Arg114
PLSK (of Combitope)	P7-Pro					
	P8-Leu					Trp147, Ala152, Gln156
	P9-Ser					Asp77, Trp147
	P10-Lys	O	Trp147	NE1 (HB)	2.93	Asp77, Thr80, Leu81, Tyr84, Ile95, Asp116, Thr143, Lys146, Trp147
		N	Asp77	OD1 (HB)	2.93	
		NZ	Asp116	OD2 (SB)	2.72	
		OT1	Tyr84	OH (HB)	2.61	
			Thr143	OG1 (HB)	2.76	
		OT2	Lys146	NZ (HB)	2.75	

[a]The cutoff distance of a hydrogen bond (HB) is 3.4 Å.

[b]The cutoff distance for salt bridge (SB) interactions is 4 Å.

[c]The cutoff distance for van der Waals (vdW) contact is 4.0 Å.

[a, b, c] The distances for various interactions were determined using CONTACT from CCP4i suite and COOT.
Residues having any atom within this distance are considered to be contact residues.
